# Navigating the Incidence of Postoperative Arrhythmia and Hospitalization Length: The Role of Amiodarone and Other Antiarrhythmics in Prophylaxis

**DOI:** 10.7759/cureus.57963

**Published:** 2024-04-10

**Authors:** Chetan Yarlagadda, Mohamed A Abutineh, Rohan R Datir, Levi M Travis, Rohan Dureja, Akshay J Reddy, Jacqueline M Packard, Rakesh Patel

**Affiliations:** 1 Medicine, Miller School of Medicine, University of Miami, Miami, USA; 2 Medicine, Edward Via College of Osteopathic Medicine, Spartanburg, USA; 3 Medicine, California University of Science and Medicine, Colton, USA; 4 Biomedical Sciences, Palm Beach State College, Boca Raton, USA; 5 Internal Medicine, Quillen College of Medicine, East Tennessee State University, Johnson City, USA

**Keywords:** pre-exposure prophylaxis, pharmacological prophylaxis, cardiology, postoperative arrhythmias, antiarrhythmics, amiodarone

## Abstract

Antiarrhythmic drugs play a pivotal role in managing and preventing arrhythmias. Amiodarone, classified as a class III antiarrhythmic, has been used prophylactically to effectively prevent atrial fibrillation postoperatively in cardiac surgeries. However, there is a lack of consensus on the use of amiodarone and other antiarrhythmic drugs as prophylaxis to reduce the occurrence of all types of postoperative arrhythmias in cardiac and non-cardiac surgeries. A comprehensive PubMed query yielded 614 relevant papers, of which 52 clinical trials were analyzed. The data collection included the class of antiarrhythmics, timing or method of drug administration, surgery type, type of arrhythmia and its incidence, and hospitalization length. Statistical analyses focused on prophylactic antiarrhythmics and their respective reductions in postoperative arrhythmias and hospitalization length.

Prophylactic amiodarone alone compared to placebo demonstrated a significant reduction in postoperative arrhythmia incidence in cardiac and non-cardiac surgeries (24.01%, p<0.0001), and it was the only treatment group to significantly reduce hospitalization length versus placebo (p = 0.0441). Prophylactic use of class 4 antiarrhythmics versus placebo also demonstrated a significant reduction in postoperative arrhythmia incidence (28.01%, p<0.0001), and while there was no significant statistical reduction compared to amiodarone (4%, p=0.9941), a lack of abundant data provides a case for further research on the prophylactic use of class 4 antiarrhythmics for this indication.

Amiodarone prophylaxis remains a prime cornerstone of therapy in reducing postoperative arrhythmia incidence and hospitalization length. Emerging data suggests a need for a broader exploration of alternative antiarrhythmic agents and combination therapies, particularly class 4 antiarrhythmics, in both cardiac and non-cardiac surgeries. This meta-analysis depicts the effectiveness of amiodarone, among other antiarrhythmics, in postoperative arrhythmia incidence and hospitalization length reduction in cardiac and non-cardiac surgeries.

## Introduction and background

Class I, II, III, and IV antiarrhythmics

Antiarrhythmic drugs encompass four main classes, each characterized by distinct mechanisms of action. Class 1A agents, such as procainamide and quinidine, block sodium channels during depolarization, slowing conduction velocity and prolonging action potential duration. Class 1B agents, such as lidocaine and mexiletine, also act on sodium channels but have no effect on conduction velocity and may shorten the action potential duration. Flecainide and propafenone, representing class 1C antiarrhythmics, exert their effects by blocking sodium channels and prolonging action potential duration. Beta-adrenergic blockers (class II), including atenolol, metoprolol, and propranolol, modulate sympathetic stimulation and myocardial contractility. Class III antiarrhythmics, including amiodarone, dofetilide, ibutilide, and sotalol, prolong the action potential through potassium channel blockade [1]. Lastly, calcium channel blockers (class IV), like verapamil and diltiazem, impede calcium influx, result in diminished myocardial contractility, and slowed atrioventricular node conduction.

Although primarily a class III antiarrhythmic, sotalol exhibits some class II beta-adrenergic blocking effects in addition to its potassium channel blockade. This dual mechanism contributes to its antiarrhythmic properties by reducing sympathetic stimulation and prolonging repolarization. Ibutilide and dofetilide are considered pure class III antiarrhythmics, as they predominantly block potassium channels, specifically the rapid component of the delayed rectifier potassium current (IKr). This results in prolonged repolarization and an increased refractory period, making them effective in managing atrial fibrillation (AFib) and atrial flutter [2].

Utility of amiodarone

Amiodarone, while classified as a class III antiarrhythmic due to its prominent potassium channel blockade, is unique and an especially powerful antiarrhythmic in that it also shares characteristics with classes I, II, and IV. It inhibits sodium channels (class I), exerts beta-adrenergic blocking effects (class II), and inhibits calcium channels (class IV). Amiodarone's pharmacological profile, characterized by its class-sharing properties across multiple antiarrhythmic classes (classes I, II, III, and IV), renders it a potent and versatile agent for the management of certain arrhythmias, particularly AFib, where both rate and rhythm control are essential [2].

Oral amiodarone can be used to treat atrial arrhythmias, but it is primarily used to maintain normal sinus rhythm in patients with AFib. Despite its common use for this indication, it is not FDA-approved for rhythm control in AFib. Oral loading doses typically range from 400 to 1200 mg/day in divided doses. The maintenance dose is usually 200 mg daily but can be as low as 100 mg daily. Intravenous (IV) amiodarone is used for the restoration and maintenance of atrial arrhythmias in critically ill patients with hemodynamically unstable AFib. An initial loading dose of 150 mg is given over at least 10 minutes, with a more rapid infusion increasing the risk of hypotension. The initial loading dose should be followed by a continuous 1 mg/minute infusion for six hours and 0.5 mg/minute after. It should be noted that multiple dosing schemes exist for the use of amiodarone [3]. Additionally, amiodarone is utilized for life-threatening ventricular arrhythmias, with an initial loading dose given intravenously and a subsequent maintenance infusion [4]. Prophylactically, it may be used to prevent AFib in post-cardiac surgery patients [5]. In certain refractory cases of supraventricular tachycardias, amiodarone may be considered, with dosing strategies tailored to the specific clinical scenario.

Postoperative arrhythmias

Postoperative arrhythmias can manifest as a result of various surgical interventions, either triggering recurrent arrhythmias or leading to new-onset rhythm disturbances [6]. The stress and physiological alterations associated with surgery create an environment conducive to arrhythmogenesis. Cardiac surgeries, especially those involving the atria or ventricles, carry an inherent risk of arrhythmias during the postoperative phase. Procedures like coronary artery bypass grafting (CABG) or valve surgeries can disrupt the normal electrical conduction system, potentially leading to AFib or ventricular arrhythmias. Non-cardiac surgeries may also contribute, with factors such as electrolyte imbalances, sympathetic activation, and inflammation playing roles in arrhythmia development [6]. Amiodarone, with its dual capacity for rate and rhythm control, assumes significance in the postoperative setting, where maintaining a stable cardiac rhythm is paramount.

The administration of amiodarone in the perioperative period has been shown to significantly reduce the occurrence of new-onset AFib in patients undergoing CABG and valvular surgeries [7]. The drug's impact on delaying repolarization and increasing the effective refractory period is thought to stabilize the cardiac rhythm during the vulnerable postoperative period. Dosing regimens vary among studies, but commonly, a loading dose of amiodarone is administered either preoperatively or intraoperatively, followed by a maintenance dose in the postoperative period [5]. The duration of prophylactic treatment may extend for several days, depending on the specific surgical procedure and individual patient characteristics.

Despite its proven efficacy, the use of amiodarone in this context necessitates careful consideration of potential side effects and complications associated with prolonged administration. Pulmonary and thyroid toxicities, among other adverse effects, underscore the importance of vigilant monitoring when utilizing amiodarone for postoperative AFib prevention [8].

While amiodarone prophylaxis to prevent AFib postoperatively in cardiac surgeries has been studied, amiodarone prophylaxis has not been extensively researched for other arrhythmias, non-cardiac surgeries, or in comparison with the use of other antiarrhythmics. This review aims to explore the usage of amiodarone in all types of surgeries and arrhythmias to assess if it has more broad prophylactic abilities than previously understood and to see if another class of antiarrhythmics could provide either synergistic effects or better outcomes on its own. Additionally, exploring the potential correlation between prophylactic amiodarone use and reduced hospital stays is essential, as early intervention to prevent postoperative arrhythmias may contribute not only to cardiovascular stability but also to expedited recovery, potentially minimizing overall hospitalization duration and minimizing the healthcare cost burden on patients.

## Review

Methods

**Figure 1:**
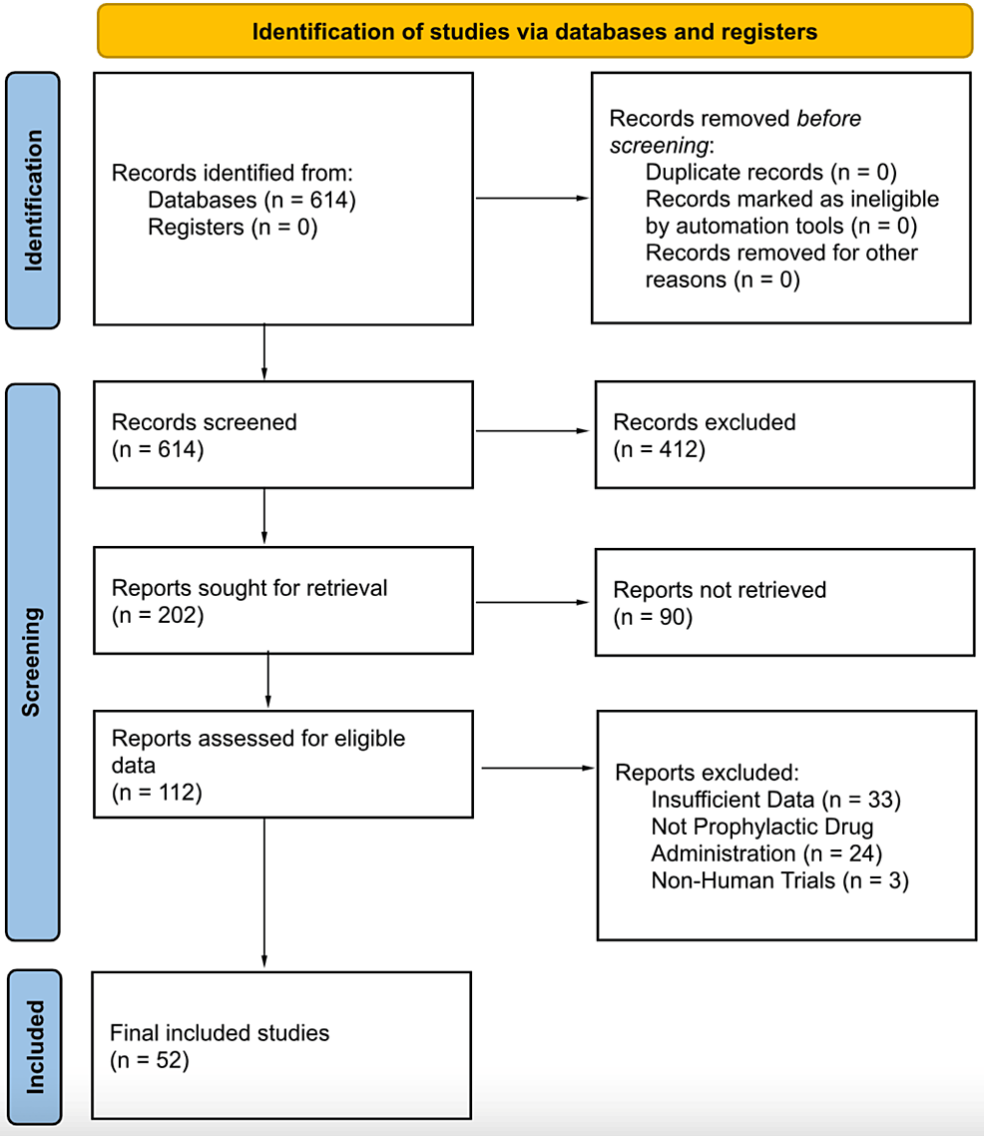
PRISMA diagram which depicts the identification, appraisal, and screening process PRISMA: Preferred Reporting Items for Systematic Reviews and Meta-Analyses

A query was conducted in PubMed using the boolean statement “amiodarone and postoperative arrhythmia” to provide the most number of studies related to the use of amiodarone in the context of postoperative arrhythmias. The process of identification consisted of using different boolean statements and operators until the most relevant papers were populated, resulting in a total of 614 papers. The authors decided that clinical trials, or randomized controlled trials (RCTs), were best for this paper as they would prove to be the most standardized approach to the real-world pharmacologic application of surgeries that are generally hard to standardize in non-clinical trials. Upon the addition of the clinical trials/RCTs criteria, 412 articles were removed, which yielded 202 clinical trials/RCTs. To remove unavailable papers, the criterion of full-text availability was added, which provided a reduction of 90 papers, bringing the total down to 112 papers that fulfilled both criteria,i.e., a clinical trial or RCT available with full text. Figure 1 demonstrates the chronological application of the aforementioned process of identifying and screening the studies.

The citations of the 112 papers were exported to Mendeley Reference Manager (Elsevier Inc., New York City, NY, USA) and were then individually screened by two authors for the relevance of each study to this meta-analysis. Major exclusion criteria that disqualified papers from being used in this study include the use of amiodarone for treating arrhythmias rather than prophylaxis, usage in animals and not humans, if the postoperative arrhythmia incidence rate was not included, or if the paper was not able to be found. Other exclusion criteria were identified on a case-by-case basis and were discussed between the two individual authors, such as whether or not there was a true comparator group to the amiodarone treatment group. Statistical analyses and the creation of the graphs were completed using the Prism 10 software (GraphPad Software Inc., La Jolla, CA, USA). Since this study required a multiple comparison test between more than two treatment groups, a one-way ANOVA was completed for both the incidence percentages of postoperative arrhythmias as well as the hospital length data. Outliers were identified by the Prism software and were removed objectively based on the inputted data. 

Common arrhythmias after cardiac surgery

Cardiac surgeries pose tremendous pharmacologic, ischemic, electrolyte, and inflammatory stress on the electrical circuit of the heart, leading to an increased incidence of arrhythmias after such surgeries. Among the most common arrhythmias after cardiac surgeries are supraventricular tachyarrhythmias, of which AFib incidence rates can be as high as 40% after a CABG, 50% after a valvular surgery, or even 60% in a valvular and CABG surgery combined [9]. While AFib is known to occasionally resolve without interventions, one study found that of the 32.3% of patients who developed AFib after CABG surgery, there was a notably high recurrence rate in about 42.6% of the patients who developed AFib [10]. Recurrent bouts of AFib can precipitate deleterious outcomes such as thromboembolic stroke, heart failure, myocardial infarction (MI), and even pulmonary embolism (PE) [11]. A total of 52 papers were analyzed in Table 1 [12-61], and 47 of them studied postoperative arrhythmias specifically after cardiac surgery [12,14-33,35-45,47-52,54,55,57-61]. Of those 47, there were 44 that studied supraventricular tachyarrhythmias, ultimately constituting 84.6% of the total studies analyzed [12,14-30,32,33,35-37,39-45,47-50,52,54,55,57-62]. Further complicating the impact of postoperative AFib is the extended length of hospital stay, which proves to be an indicator of increased healthcare expenditures as well as patient morbidity [62]. 

**Table 1:** Postoperative arrhythmia incidence rates and hospitalization length with amiodarone and other antiarrhythmics w/: With; MRS: Myocardial revascularization surgery; CABG: Coronary artery bypass graft; CHD: Congenital heart defect; CTS: Cardiothoracic surgery; AFib: Atrial fibrillation; IV: Intravenous, VTach: Ventricular tachycardia; VFib: Ventricular fibrillation; N/A: Not available; SPPAF: Study of prevention of postoperative atrial fibrillation; CAMIAT: Canadian Amiodarone Myocardial Infarction Arrhythmia Trial; AFIST: Atrial Fibrillation Suppression Trial; PAPABEAR: Prophylactic amiodarone for the prevention of arrhythmias that begin early after revascularization, valve replacement, or repair; ARCH: Amiodarone reduction in coronary heart; REDUCE: Reduction in postoperative cardiovascular arrhythmic events

Trial and/or authors (year)	Amiodarone, treatment(s) in conjunction	Comparator treatment(s)	Timing of administration	Method of administration	Sample size	Surgery type	Postoperative arrhythmia, incidence percentage	Length of hospital stay (days)
Alcalde et al. (2006) [12]	Amiodarone	Placebo	Started 30 to 56 hours preoperatively	Oral	93	MRS	AFib or AFlutter, 17.4% of amiodarone group vs 40.4% of comparator group	8.9 w/ treatment vs 11.5 w/ comparator
Amar et al. (2022) [13]	Amiodarone w/ N-acetylcysteine	Amiodarone w/ Placebo	Started postoperatively and continued for 48 hours	Intravenous	154	Elective major thoracic surgery	AFib, 19% of treatment group versus 17% of comparator group	5 w/ treatment vs 6 w/ comparator
Amrousy et al. (2016) [14]	Amiodarone	Placebo	Started 0.5 hours preoperatively	Intravenous	117	CHD surgery	Junctional ectopic tachycardia, 9.2% of amiodarone group vs 28.9% of comparator group	7.8 w/ treatment vs 9.3 w/ comparator
SPPAF, Auer et al. (2004) [15]	Amiodarone w/ metoprolol	Metoprolol only, sotalol only, or placebo only	Started 24 to 48 hours preoperatively	Oral	253	CABG and/or valvular surgery	AFib, 30.2% of treatment group vs 31.7% of sotalol group vs 53.8% of placebo group	11.3 w/ all medication groups vs 13.1 w/ placebo
Bockeria et al. (2020) [16]	Amiodarone-releasing hydrogel	Placebo	Started intraoperatively	Spray	60	CABG	AFib, 3.3% for treatment group vs 37% for comparator group	6 for treatment group vs 8 for comparator group
Budeus et al. (2006) [17]	Amiodarone	Placebo	Started 24 hours preoperatively	Oral, but intravenous for intraoperative administration	110	CABG	AFib, 34% for treatment group vs 85% for comparator group	11.3 for treatment group vs 13 for comparator group
Butler et al. (1993) [18]	Amiodarone	Placebo	Started 24 hours preoperatively	Intravenous, then oral postoperatively	120	CABG	Afib, 8% in treatment group vs 20% in comparator group	Median 7 for both conditions
Cagli et al. (2006) [19]	Amiodarone w/ magnesium sulfate	Amiodarone only or saline only	Started as soon as possible postoperatively in the ICU	Intravenous	136	CABG	AFib, 9.1% for treatment group vs 36.4% for amiodarone only group vs 33.3% for saline only group	N/A
Carter et al. (2012) [20]	Amiodarone	No drug	Started the night before surgery, approximately 8 to 24 hours preoperatively	Oral	198	CTS	AFib, 23% for treatment group vs 43% for comparator group	6.8 for treatment group vs 8.0 for comparator group
CAMIAT, Crystal et al. (2003) [21]	Amiodarone	Placebo	Started up to 8.5+ months pre-operatively then discontinued within 84 hours of surgery	Oral	82	CTS	AFib, 16.7% for treatment group vs 24.8% for comparator	6.1 for treatment group vs 3.6 for comparator group
Daoud et al. (1997) [5]	Amiodarone	Placebo	Started 172 hours preoperatively	Oral	124	CTS	AFib, 25% for treatment group vs 53% for comparator group	6.5 for treatment group vs 7.9 for comparator group
Delle Karth et al. (2007) [22]	Amiodarone	Placebo	Started 84 hours preoperatively	Oral, then intravenous	22	CTS	AFib, 33.3% for treatment group vs 37.5% for comparator group	9 for treatment group vs 10 for comparator group
Dimopoulou et al. (1997) [23]	Amiodarone	No drug	Started preoperatively. Varies by patient. Ranges from 7 to 1440 days.	N/A	88	CTS	AFib, 13.6% in treatment group vs 4.5% in comparator group. VTach, 0% in treatment group vs 2.3% in comparator group	8 for both treatment group and comparator group
Dörge et al. (2000) [24]	Amiodarone	Placebo	Started postoperatively and continued for 72 hours	Intravenous	150	CTS	AFib, 24% for treatment group 1 vs 28% for treatment group 2 vs 34% for comparator group	14.0 for treatment group 1 vs 14.4 for treatment group 2 vs 14.7 for comparator group
Feng et al. (2014) [25]	Poly-based hydrogel w/ amiodarone	Poly-based hydrogel w/ no drug	Started intraoperatively	Spray	100	CTS	AFib, 8% for treatment group vs 26% for comparator group	N/A
AFIST, Giri et al. (2001) [7]	Amiodarone	Placebo	Started 24 to 120 hours preoperatively	Oral	220	CTS	AFib, 22.5% for treatment group vs 38.0% for comparator group	N/A
ARCH, Guarnieri et al. (1999) [26]	Amiodarone	Placebo	Started immediately post operation, continued for 48 hours	Intravenous	300	CTS	AFib, 35% for treatment group vs 47% for placebo	7.6 for treatment group vs 8.2 for placebo
Halonen et al. (2010) [27]	Amiodarone	Metoprolol	Started postoperatively, continued for 48 hours	Intravenous	316	CTS	AFib, 24.8% for treatment group vs 23.9% for comparator group	5.4 for treatment group vs 5.6 for comparator group
Hohnloser et al. (1991) [28]	Amiodarone	Placebo	Started immediately post operation, followed by 96 hours of administration	Intravenous (infusion via central line)	77	CABG	AFib, 5% in treatment group vs 21% in comparator group	N/A
Kar et al. (2011) [29]	Amiodarone	Placebo	Started 0.33 hours preoperatively	Intravenous	56	Valve Replacement Surgery	AFib, 7.1% for treatment group vs 28.6% for comparator group; VTach/VFib, 21.4% for treatment group vs 46.4% for comparator group	6 for treatment group vs 6 for comparator group
GAP, Kerstein et al. (2004) [30]	Amiodarone	Placebo	Started preoperatively 120 hours before surgery, then continued 3 to 5 days post-operation	Intravenous, then oral	51	CABG	AFib, 5.9% for treatment group vs 26.1% for comparator group	5.3 for treatment group vs 6.7 for comparator group
AFIST, Kluger et al. (2003) [31]	Amiodarone w/ beta blockers	Beta blockers	Started 24 to 120 hours preoperatively	Oral	220	CTS	VTach, 1.7% of treatment group vs 7% of comparator group. AFib, 23% of treatment group vs 38% of comparator group.	N/A
Kojuri et al. (2009) [32]	Amiodarone w/ Propranolol	Amiodarone only or Propranolol only	Started administration 192 hours preoperatively, continued for 120 hours post-operation	Oral	240	CABG	AFib, 5% of amiodarone and propranolol group vs 6.3% of amiodarone group vs 16.3% of propranolol group	N/A
Kuralay et al. (2004) [33]	Amiodarone	No drug	Started IV immediately post-operation	Intravenous	200	CABG	AFib, 12% in treatment group vs 28% in comparator group	14.5 for treatment group vs 17.6 for comparator group
Lanza et al. (2003) [34]	Amiodarone	No drug	Started postoperatively and continued until discharge	Oral	83	Pulmonary resection by thoracotomy	AFib, 9.7% for treatment group vs 33% for comparator group	6 for treatment group vs 7 for comparator group
Lee et al. (2000) [35]	Amiodarone	Placebo	Started IV bolus 72 hours before the operation, continued for five days post-operation	Intravenous	150	CABG	AFib, 12% in treatment group vs 34% in comparator group	17 for treatment group vs 19 days for comparator group
Maras et al. (2001) [36]	Amiodarone	Placebo	Started 24 hours before surgery, continued for 7 days post-operation	Oral	315	CABG	AFib, 19.5% for treatment group vs 21.2% for comparator group	10.3 for treatment group vs 10.4 for comparator group
Mikroulis et al. (2005) [37]	Amiodarone	Diltiazem only or beta blocker only	Started 48 hours preoperatively, followed by postoperative beta blocker until discharge	Intravenous	180	CABG	AFib, 11.7% for amiodarone group vs 10% for diltiazem group vs 23.3% for beta blocker group	N/A
Mita et al. (2019) [38]	Amiodarone	Lidocaine	Started preoperatively the morning of the surgery	Intravenous	68	Aortic valve replacement	VFib, 20.6% for treatment group vs 50% for comparator group	23 for treatment group vs 24 for comparator group
PAPABEAR, Mitchell et al. (2005) [39]	Amiodarone	Placebo	Started preoperatively for 144 hours, continued for 144 hours post-operation	Oral	601	Revascularization and/or valvular Surgery	Atrial tachyarrhythmia, 16.1% for treatment group vs 29.5% for comparator group	8.2 for treatment group vs 8.9 for comparator group
REDUCE, Mooss et al. (2004) [40]	Amiodarone	Sotalol	Started immediately preoperatively, continued for 168 hours post-operation	Intravenous, then oral	160	CABG and/or aortic valve replacement surgery	AFib, 17% for treatment group vs 25% for comparator group	5.5 for treatment group vs 6.0 for comparator group
Nygård et al. (2004) [41]	Amiodarone	Placebo	Started day of surgery, continued for 48 hours postoperatively	Intravenous	165	CABG	AFib, 27.8% for treatment group vs 41.7% for comparator group	7 for treatment group vs 8 for comparator group
Onk et al. (2015) [42]	Amiodarone	Metoprolol	Started preoperatively 168 hours surgery	Oral	251	CABG	AFib, 11.5% for treatment group vs 12.4% for comparator group	7.9 for treatment group vs 8.1 for comparator group
Pong et al (2020) [43]	Amiodarone	No Drug	Started intraoperatively	Intravenous	57	CABG and/or Valvular Surgery	Afib, 25% for treatment group vs 68% for comparator group	7.0 for treatment group vs 8.2 for comparator group
Reddy et al. (2004) [44]	Amiodarone	Placebo only or amiodarone w/ pacing	Started 6 hours preoperatively followed by oral administration for next 24 to 96 hours postoperatively	Intravenous, then oral	160	CTS	AFib, 28% for amiodarone group vs 38% for placebo group vs 16% for amiodarone + pacing group	N/A
Redle et al. (1999) [45]	Amiodarone	Placebo	Started postoperatively for 7 days	Oral	143	CABG	AFib, 24.7% in treatment group vs 32.8% in comparator group	N/A
Riber et al. (2012) [46]	Amiodarone	Placebo	IV started immediately post-operation, followed by oral dose for120 hours post-operation	Intravenous, then oral	254	Lung cancer surgery	AFib, 9.0% for treatment group vs 31.7% for comparator group	N/A
Roshanali et al. (2009) [47]	Amiodarone	Placebo	Started preoperatively followed by IV administration during surgery and the day after surgery, followed by oral administration up to 120 hours postoperatively	Oral, then intravenous, then oral again	100	CABG	AFib, 16% for treatment group vs 88% for comparator group	5.6 for treatment group vs 7.8 for comparator group
Skiba et al. (2013) [48]	Amiodarone w/ metoprolol	Metoprolol only or no placebo only	Started IV administration intraoperatively	Intravenous	215	CTS	AFib, 22% for amiodarone w/ metoprolol group vs 35% for metoprolol only group vs 34% for placebo group	6 for all conditions
Sleilaty et al. (2009) [49]	Amiodarone	Bisoprolol	Started 6 hours postoperatively	Oral	200	CABG	AFib, 15.3% for treatment group vs 12.7% for comparator group	6 for treatment group vs 5.5 for comparator group
Solomon et al. (2001) [50]	Amiodarone	Propanolol	Started within three hours preoperatively	Intravenous, then oral	102	CTS	AFib, 16.0% in treatment group vs 32.7% in comparator group	8.8 for treatment group vs 8.4 for comparator group
Som et al. (2017) [51]	Amiodarone	Dronedarone	Started 0.33 hours preoperatively	Intravenous	36	CABG	Any arrhythmia, 50% for treatment group vs 16.7% for comparator group	6.5 for treatment group vs 6 for comparator group
Stamou et al. (2001) [52]	Amiodarone	Placebo	Started postoperative administration up to 168 hours	Oral	1196	CTS	AFib, 25% for treatment group vs 31% for comparator group	N/A
Tisdale et al. (2010) [53]	Amiodarone	No drug	Started preoperatively during anesthesia induction, continued for a total of 96 hours postoperatively	Intravenous	80	Transthoracic esophagectomy	AFib, 15% of treatment group vs 40% of comparator group	11 days w/ treatment vs 12 days w/ comparator
Tokmakoglu et al. (2002) [54]	Amiodarone	Digitoxin + metoprolol or no drug	Started one-hour post-operation	Intravenous	241	CABG	AFib, 8.3% for amiodarone group vs 16.8% for digitoxin + metoprolol group vs 33.6% for control group	N/A
Treggiari-Venzi et al. (2000) [55]	Amiodarone	Magnesium only or placebo only	Started postoperatively and continued for 72 hours	Intravenous	155	CABG	AFib, 14% for amiodarone group vs 23% for magnesium group vs 27% for placebo group	4 for amiodarone vs 3 for magnesium vs 3 for placebo
Van Mieghem et al. (1994) [56]	Amiodarone	Verapamil only or placebo only	Started postoperatively one hour after surgery	Intravenous	96	Lobectomy or pneumonectomy	AFib, lobectomy: 4.8% in amiodarone group vs 0% in verapamil vs 27.2% in placebo group. Pneumonectomy: 0% in amiodarone group vs 0% in verapamil vs 10% in placebo group	N/A
Wang et al. (2016) [57]	Poly-based hydrogel w/ amiodarone	Hydrogel spray only (no drug)	Started intraoperatively	Spray	150	CABG	AFib, 8% for treatment group vs 26% for comparator group	N/A
AFIST II, White et al. (2003) [58]	Amiodarone	Placebo	Started preoperatively within six hours of surgery, continued for 96 hours postoperatively	Intravenous, then oral	160	CTS	AFib, 22.1% in treatment vs 38.6% in comparator	7.9 for treatment group vs 11.4 for comparator group
Yagdi et al. (2003) [59]	Amiodarone	Placebo	Started preoperatively within two hours, continued postoperatively for 48 hours	Intravenous, then oral	157	CABG	AFib, 10.4% in treatment group vs 25.0% in comparator group	6.8 days for treatment group vs 7.8 days for comparator group
Yazigi et al. (2002) [60]	Amiodarone	Placebo	Started postoperatively and continued until discharge	Oral	200	Coronary artery surgery	AFib, 12% for treatment group vs 25% for comparator group	6.8 days for treatment group vs 7.1 days for comparator group
Zebis et al. (2007) [61]	Amiodarone	Placebo	Started postoperatively and continued for 120 hours	Intravenous, then oral	250	CABG	AFib, 12% for treatment group vs 29% for comparator group	13.8 for treatment group vs 13.5 for comparator group

While less common, bradyarrhythmias and ventricular tachyarrhythmias are at increased odds of precipitating after cardiac surgery and pose a significant mortality risk. Ventricular tachyarrhythmias in particular are a rare but deadly presentation of postoperative arrhythmias, and it has been noted that patients with sustained ventricular arrhythmias have a mortality rate due to cardiac issues of up to 20% [9]. Of the 47 studies in Table 1 that used cardiac surgeries as the surgery type, five papers studied the effects of ventricular tachyarrhythmias, comprising only 9.62% of the total studies analyzed [23,29,31,38,51]. Prophylaxis of supraventricular arrhythmias with amiodarone has been studied, and there has been data in support of such a use, but unfortunately, there is not a general consensus on the efficacy of amiodarone prophylaxis for postoperative non-supraventricular tachyarrhythmias [6]. Though ventricular arrhythmias are not as common after cardiac surgery, the potentially high-risk consequences of their incidence are not to be ignored.

Postoperative arrhythmias in non-cardiac surgeries

Interestingly, non-cardiac surgeries present with notable increases in postoperative arrhythmias, albeit on a smaller scale than cardiac surgeries, but present a unique challenge to the management of patients in the postoperative period. Although direct stress is not placed on the heart, a systemic inflammatory and sympathetic challenge is placed on the cardiovascular system even in non-cardiac surgeries, which can predispose susceptible patients to arrhythmias postoperatively [5]. Five studies in this analysis, as noted in Table 1, studied the effects of non-cardiac thoracic surgeries and their incidence of postoperative arrhythmias, and all five studies focused on AFib in particular [13,34,46,53,56]. Newer data supports the theory that non-cardiac surgeries do exhibit an increased incidence of postoperative arrhythmias, but unfortunately, the same breadth of data that exists for cardiac surgeries is not yet available for non-cardiac surgeries [63]. A recent cohort study found that certain thoracic surgeries had a remarkably high rate of AFib, such as pancreatectomies (16.7% of patients), elective thoracoabdominal esophagectomies (17.1% of patients), and emergency esophagectomies (45.5% of patients) [64]. It was theorized that those aforementioned surgeries, among others in the study, precipitated AFib at such rates due to their highly invasive nature. In particular, the emergency esophagectomies done due to a perforation likely caused hemodynamic instability and a remarkably high inflammatory response, contributing to a proarrhythmic cardiac state and causing high rates of postoperative arrhythmias [64]. While the data is sparse regarding postoperative arrhythmias in non-cardiac surgeries, this review could shed light on not only the optimal pharmacologic prophylaxis of postoperative supraventricular and ventricular arrhythmias, but more importantly, in both cardiac and non-cardiac surgeries.

Prophylactic pharmacologic interventions for postoperative arrhythmias

The statistical analysis conducted in this review, as seen in Table 2, is consistent with the notion that pharmacologic antiarrhythmic prophylaxis before cardiac and non-cardiac surgeries is a significant factor in reducing the incidence of postoperative arrhythmias. Amiodarone continued to be an effective prophylactic measure in comparison to placebo in reducing the average incidence of postoperative arrhythmias (Table 2), with only 7.33% incidence of postoperative arrhythmias versus the 31.34% of patients that developed an arrhythmia in the placebo group. In combination with amiodarone, class 2 antiarrhythmics, which consist of beta blockers, have also been shown (Table 2) to reduce the incidence of postoperative arrhythmias compared to placebo. Beta blockers are a commonly used antiarrhythmic and have been previously studied as a prophylactic agent to be effective in reducing the incidence of postoperative arrhythmias such as AFib in specific surgeries [65]. However, it is important to take into account other systemic effects of beta blockers, such as hypotension and bradycardia, which have been associated with a greater rate of total mortality despite having lower rates of cardiac events when administered in the perioperative period [65]. Furthermore, determining the right beta blocker to optimally reduce postoperative AFib can heavily influence outcomes. Though selection should be personalized to the given patient, carvedilol is associated with a significant decrease in postoperative AFib, potentially owing to its additive effect of decreasing cardiac afterload through alpha blockade [66]. In this study, class 2 antiarrhythmics alone did not yield a statistically greater reduction in postoperative arrhythmias (p = 0.086); however, the combination therapy of amiodarone and beta blockers provided a statistically significant postoperative arrhythmia reduction of 13.71% in comparison to placebo. By itself, amiodarone provided a significant reduction in comparison to 24.01% in the placebo group. While not statistically greater in comparison to the amiodarone with beta blocker condition, it may be a clinically significant reduction in comparison.

**Table 2:** Statistically significant findings of the differences in postoperative arrhythmia incidence per treatment intervention group w/: With, C2: Class 2, C4: Class 4

Treatment comparison	Mean 1 (%)	Mean 2 (%)	Mean difference (%)	Adjusted p-value
Amiodarone vs placebo	7.33	31.34	-24.01	<0.0001
Amiodarone w/ C2 antiarrhythmic vs placebo	17.63	31.34	-13.71	0.0097
C4 antiarrhythmic vs placebo	3.33	31.34	-28.01	<0.0001
Amiodarone w/ non-antiarrhythmic vs placebo	14.7	31.34	-16.64	0.0336
C4 antiarrhythmic vs C2 antiarrhythmic	3.33	22.59	-19.26	0.0207

Three studies (Table 1) used amiodarone in conjunction with non-antiarrhythmics, including N-acetylcysteine (NAC), magnesium sulfate, or cardiac pacing. This group, while also statistically greater than the placebo group in reducing the incidence of postoperative arrhythmias, faces a similar issue to the amiodarone with beta blockers group in that they both are not significantly greater than amiodarone by itself and in fact yield worse incidence rates. Though NAC in conjunction with amiodarone did not yield a statistically significant reduction in incidence rates, it has been shown to do so as a single agent in previous studies, including a prospective, randomized, double-blind study of 115 patients undergoing CABG in which postoperative AFib rates were reduced by 16% [67]. It has also reduced such incidence rates in conjunction with carvedilol in comparison to placebo, carvedilol alone, and metoprolol [68]. However, the evidence for such a reduction is conflicting, as a 2011 meta-analysis of 15 RCTs with 1,407 patients showed NAC during cardiac surgery did not significantly decrease postoperative AFib, acute renal failure, or mortality [69]. A more recent 2019 meta-analysis corroborated these findings, analyzing 29 RCTs with 2,486 participants and demonstrating that NAC use resulted in a non-statistically significant reduction in arrhythmia or mortality [70]. Since NAC has antioxidant and anti-inflammatory properties through its ability to replenish cellular glutathione stores, theoretically it may reduce the oxidative stress associated with increased rates of AFib and other postoperative ischemic-reperfusion cardiac injury, but data at the moment does not definitely support its use [71].

Magnesium administration has had favorable data, including a meta-analysis of 20 RCTs with 2,490 patients that demonstrated a reduction of postoperative AFib from 28% in the control group to 18% in the treatment group [72]. Similarly, a more recent meta-analysis demonstrated positive effects of magnesium in smaller studies, but five double-blinded intention-to-treat studies within the meta-analysis showed magnesium did not prevent postoperative AFib [73]. Furthermore, this analysis demonstrated a non-significant dose-response relationship between magnesium dose and postoperative AFib protection. It would be beneficial to conduct larger double-blind, intention-to-treat studies to examine the effects of NAC and magnesium sulfate, as these agents are cheaper than other antiarrhythmics and have other systemic benefits. 

Interestingly, prophylaxis with class 4 antiarrhythmics, which consists of non-dihydropyridine calcium channel blockers, provided not only a statistically greater reduction in the incidence of postoperative arrhythmia than placebo but also in comparison to beta blockers. While they do not depict statistical significance in their reduction of arrhythmia incidence rates in comparison to amiodarone, they still demonstrated an incidence that is less than half of the amiodarone-only group (Table 2). Class 4 antiarrhythmic prophylaxis has sparsely demonstrated an ability to prevent supraventricular tachycardias in both cardiac and non-cardiac surgeries; however, it is not indicated for this usage [65]. A meta-analysis including 12 RCTs with 1,465 patients demonstrated a significant reduction in postoperative supraventricular tachycardia (SVT) after cardiac surgery (OR 0.62, 95% CI 0.41 to 0.93; p = 0.02) when compared to the control group [74]. However, there was significant heterogeneity among these studies, and a consistent answer is not clearly understood for the efficacy of class 4 antiarrhythmics for prophylaxis of postoperative arrhythmias.

**Figure 2:**
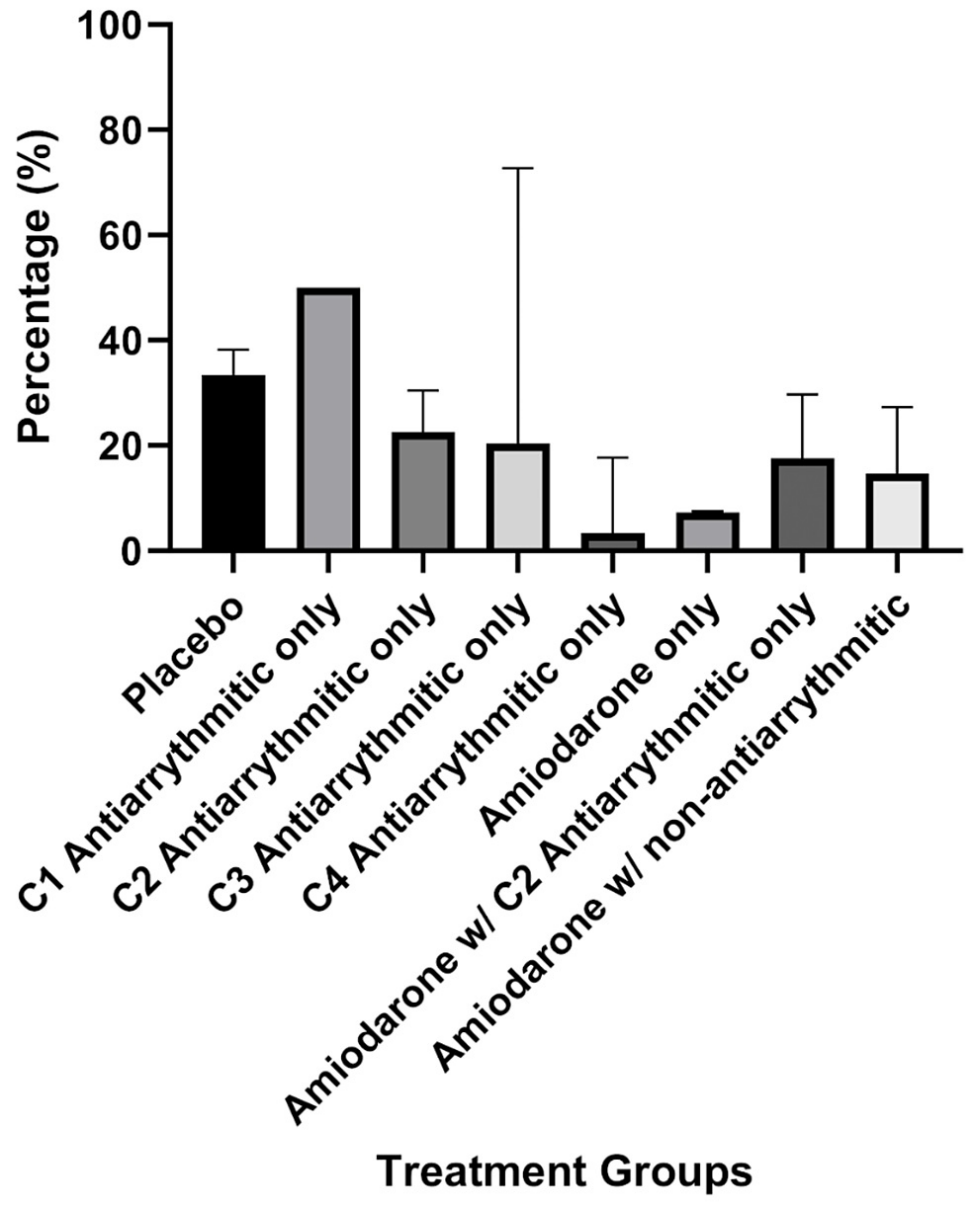
Visual comparison of the mean incidence percentages of postoperative arrhythmia per treatment group C1: Class 1, C2: Class 2, C3: Class 3, C4: Class 4

Figure 2 demonstrates the remarkably low incidence rate of postoperative arrhythmia in the class 4 antiarrhythmic group by itself in comparison to all groups studied in this review. The lack of statistical significance in this group compared to amiodarone is likely due to the lack of an abundance of studies using class 4 antiarrhythmics for the prophylaxis of postoperative arrhythmias (n = 3), as well as the intragroup variability in the incidence rates. As powerful as prophylaxis with the amiodarone-only group was in reducing postoperative arrhythmias, future studies need to investigate the prophylactic abilities of class 4 antiarrhythmics for this indication as well. Without statistical significance, a clear answer cannot be ascertained, but there is a potential investigative future in the comparative reductions of postoperative arrhythmias with amiodarone versus class 4 antiarrhythmic prophylaxis. 

Changes in length of hospital stay due to prophylactic pharmacologic agents

**Figure 3:**
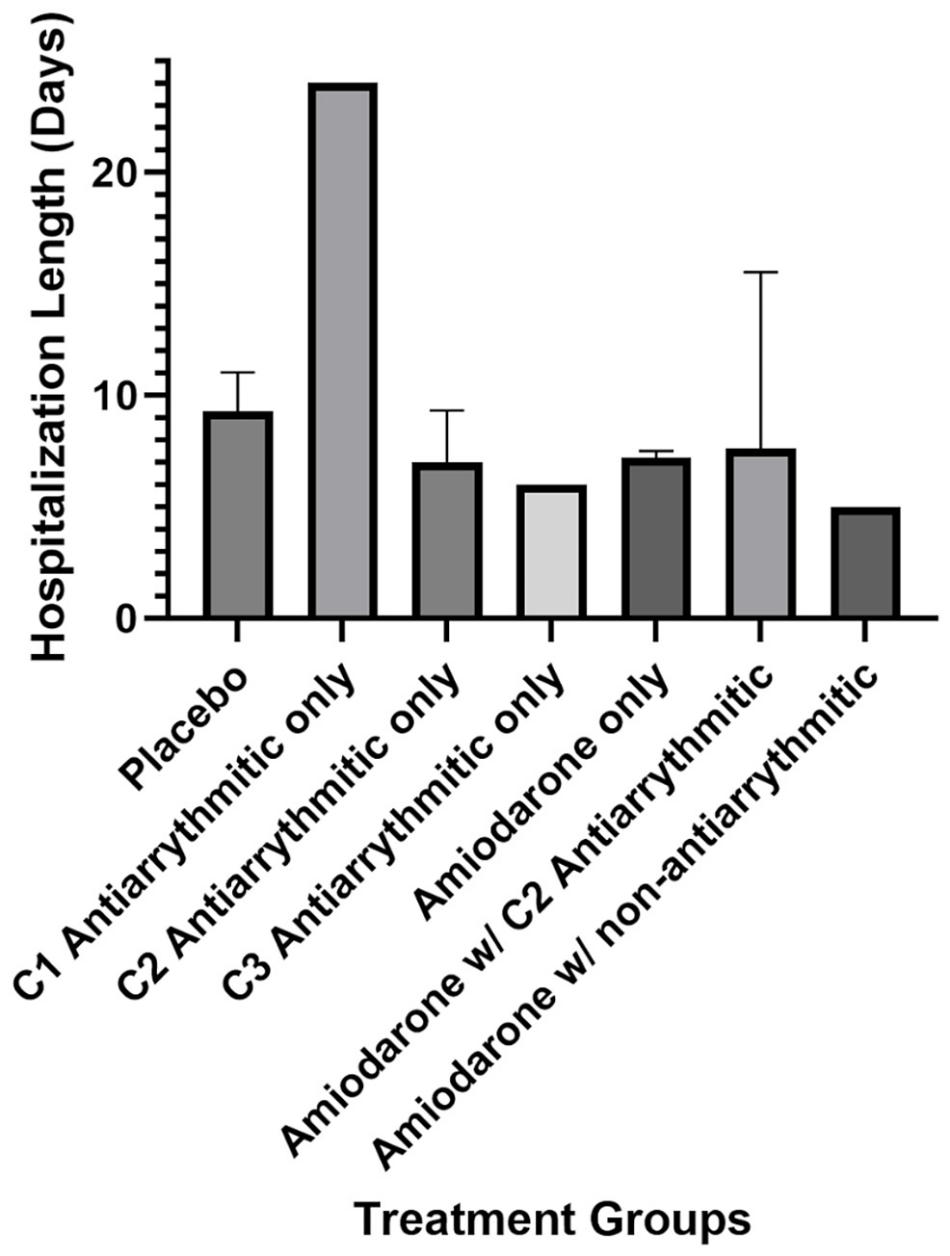
Visual comparisons of postoperative length of hospital stay (days) in each treatment group w/: With, C1: Class 1, C2: Class 2, C3: Class 3

The hospitalization length of prophylaxis for postoperative arrhythmias with antiarrhythmics was analyzed to corroborate the reduction in arrhythmia incidence with a reduction in hospital stays. When all the conditions were studied, the amiodarone-only group was the only one with a statistically significant reduction in hospital stay compared to the placebo group (p = 0.0441). A previous meta-analysis demonstrated that the prophylactic use of amiodarone significantly reduced the duration of hospital stays following cardiac surgery [75]. The analysis consisted of 19 clinical trials and found that there was an average reduction of 0.6 days in hospitalization duration. While this analysis solely focused on the usage of amiodarone after cardiac surgeries, the purpose of the current review is to indicate the effectiveness of amiodarone prophylaxis for all types of surgeries and arrhythmias. Although the other conditions are not statistically significant, investigators would be remiss to ignore that all the other conditions except for class 1 antiarrhythmics follow a trend (Figure 3) that suggests a similar reduction in hospital stay with the administration of amiodarone alone. In particular, the class 2 and 3 antiarrhythmics, not including amiodarone, as well as the amiodarone with a non-antiarrhythmic condition, all have a lower mean hospital stay. These trends, though not conclusive, hint at the possibility that, with larger, more targeted studies, other antiarrhythmics might also demonstrate benefits in the surgical context. This underscores the need for further research to solidify amiodarone's role in prophylactic care and to explore the potential of other antiarrhythmic medications in surgical recovery processes.

One glaring issue lies in the fact that many studies report arrhythmia incidence but not the length of hospital stay, especially as hospital stay is an indicator of increased mortality risk [76]. Similarly to many of the conditions in the arrhythmia incidence data, a deficit of data in hospitalization length makes it difficult to provide another pharmacologic prophylactic treatment that meets statistical significance, such as the amiodarone-only group, which has a plethora of data.

Future applications and limitations 

This comprehensive meta-analysis provides a condensed view of postoperative arrhythmias in cardiac and non-cardiac surgeries and provides clinicians with a multifaceted pharmacologic array of options to treat them. In our review, only five studies documented the use of prophylactic antiarrhythmic pharmacology in non-cardiac surgery, and those studies all used amiodarone as the primary antiarrhythmic, which restricts the ability to provide further comparisons between amiodarone and other antiarrhythmics in non-cardiac surgeries [13,34,46,53,56]. In this data set, the vast majority of surgeries (both cardiac and non-cardiac) focused on amiodarone prophylaxis without another antiarrhythmic. This meta-analysis revealed a trend towards a potential for successful prophylaxis of postoperative arrhythmias and a reduction in hospitalization lengths, leading the authors to implore future clinical trials to explore further uses of non-amiodarone antiarrhythmics, non-antiarrhythmics, or amiodarone in combination with either of the aforementioned classes of pharmacologic agents. However, while the authors were limited in certain aspects of data collection, the results were strong enough to understand the prophylactic abilities of amiodarone along with other antiarrhythmics in a multitude of surgeries. In particular, the further usage of class 4 antiarrhythmics for prophylaxis of arrhythmias postoperatively is an intriguing area, as it proved very strong reductions in incidence rates of arrhythmias, and while it was not statistically greater in reducing rates compared to amiodarone, the trend pointed towards the potential for its clinical superiority. Previous studies have investigated the use of verapamil or diltiazem, class 4 antiarrhythmics, for such a use, and the results support our statements.

Unfortunately, a multitude of shortcomings plague this topic in that the studies focused only on very specific cardiac surgeries such as CABG, and the arrhythmias studied were limited to specific arrhythmias only, namely AFib [77]. Another condition that holds potential is the cohort of patients given prophylactic amiodarone combined with a non-antiarrhythmic. While that group did not have a mean lower rate of incidence of arrhythmias compared to amiodarone, those patients demonstrated a significant reduction in incidence compared to placebo. The notable part was that the condition exhibited a large intra-group variation, but one study using amiodarone and magnesium sulfate demonstrated a lower incidence than amiodarone with class 2 antiarrhythmics [55].

Magnesium sulfate’s properties are pleiotropic in that it exhibits direct antiarrhythmic effects and mitigates states of potential electrolyte fluctuations and imbalances during invasive surgeries [5,78,79]. This perpetuates the need for a broader understanding of the many various antiarrhythmics and non-antiarrhythmic pharmacologic agents that could provide marked reductions in postoperative arrhythmias in cardiac and non-cardiac surgeries if given prophylactically. The ongoing *prophylaxis for patients at risk to eliminate post-operative atrial fibrillation* (PREP-AF) clinical trial, which is studying the usage of prophylactic amiodarone for preventing AFib in patients undergoing major non-cardiac esophageal or pulmonary surgeries, is a study to be followed [80]. Though this is just one current study, it addresses the need for further evaluation of amiodarone in non-cardiac surgeries; future clinical trials should also seek to study the efficacy of non-amiodarone antiarrhythmics for the prophylactic benefit of reducing postoperative arrhythmias. 

## Conclusions

This meta-analysis aimed to understand the efficacy of prophylactic amiodarone in comparison to other antiarrhythmics in reducing the rates of postoperative arrhythmias and hospitalization length after cardiac and non-cardiac surgeries. This review consists of 52 papers, of which five studied the effects of amiodarone in non-cardiac surgeries where seven pharmacologic agents were used prophylactically in the context of both supraventricular and ventricular arrhythmias with the intent of providing clinicians with pharmacologic tools to prevent adverse arrhythmic outcomes in high-risk surgical candidates. The data reaffirmed amiodarone to be significant in reducing postoperative arrhythmia incidence, but the other conditions that also provided significant reductions in comparison to placebo included class 4 antiarrhythmics and amiodarone combined with either class 2 antiarrhythmics or various cardioprotective non-antiarrhythmic drugs. While not statistically significant, a strong trend points towards class 4 antiarrhythmics potentially providing the best prophylactic abilities, notably in comparison to amiodarone, in which class 4 antiarrhythmics had a greater than 50% reduction.

The administration of amiodarone proved to be the only condition that provided a statistically significant reduction in hospital stay, but the data for this was sparse, and future studies exploring the use of antiarrhythmic prophylaxis in surgeries need to record the length of hospitalization. Furthermore, the data is relatively lacking on the prophylactic use of non-amiodarone antiarrhythmics as well as amiodarone therapies combined with other drugs, and future studies should seek to diversify pharmacologic treatments as this review shows the potential efficacy of other antiarrhythmic options. Though a clear antiarrhythmic was not singled out as the most optimal drug of choice, the benefit is evident in the use of prophylactic amiodarone among other antiarrhythmics in both cardiac and non-cardiac surgeries to reduce the rates of postoperative arrhythmias. Clinicians should consider amiodarone, among a few select antiarrhythmics, as a potential first-line therapy to prevent postoperative arrhythmias.
